# Modeling the Ongoing Dynamics of Short and Long-Range Temporal Correlations in Broadband EEG During Movement

**DOI:** 10.3389/fnsys.2019.00066

**Published:** 2019-11-08

**Authors:** Maitreyee Wairagkar, Yoshikatsu Hayashi, Slawomir J. Nasuto

**Affiliations:** Brain Embodiment Laboratory, Biomedical Engineering, School of Biological Sciences, University of Reading, Reading, United Kingdom

**Keywords:** Long-Range Temporal Correlation (LRTC), Short-Range Dependence (SRD), Autoregressive Fractionally Integrated Moving Average (ARFIMA), electroencephalography (EEG), Brain Computer Interface (BCI), movement intention, broadband, single trial

## Abstract

Electroencephalogram (EEG) undergoes complex temporal and spectral changes during voluntary movement intention. Characterization of such changes has focused mostly on narrowband spectral processes such as Event-Related Desynchronization (ERD) in the sensorimotor rhythms because EEG is mostly considered as emerging from oscillations of the neuronal populations. However, the changes in the temporal dynamics, especially in the broadband arrhythmic EEG have not been investigated for movement intention detection. The Long-Range Temporal Correlations (LRTC) are ubiquitously present in several neuronal processes, typically requiring longer timescales to detect. In this paper, we study the ongoing changes in the dynamics of long- as well as short-range temporal dependencies in the single trial broadband EEG during movement intention. We obtained LRTC in 2 s windows of broadband EEG and modeled it using the Autoregressive Fractionally Integrated Moving Average (ARFIMA) model which allowed simultaneous modeling of short- and long-range temporal correlations. There were significant (*p* < 0.05) changes in both broadband long- and short-range temporal correlations during movement intention and execution. We discovered that the broadband LRTC and narrowband ERD are complementary processes providing distinct information about movement because eliminating LRTC from the signal did not affect the ERD and conversely, eliminating ERD from the signal did not affect LRTC. Exploring the possibility of applications in Brain Computer Interfaces (BCI), we used hybrid features with combinations of LRTC, ARFIMA, and ERD to detect movement intention. A significantly higher (*p* < 0.05) classification accuracy of 88.3 ± 4.2% was obtained using the combination of ARFIMA and ERD features together, which also predicted the earliest movement at 1 s before its onset. The ongoing changes in the long- and short-range temporal correlations in broadband EEG contribute to effectively capturing the motor command generation and can be used to detect movement successfully. These temporal dependencies provide different and additional information about the movement.

## 1. Introduction

Movement is the primary mode of interaction with the environment and hence studying the neuronal processes involved in movement generation is interesting. The temporal and spectral changes occur in the neuronal processes during voluntary movement. Detecting the movement intention by identifying these changes in the neuronal processes observed in electroencephalography (EEG), not only helps to understand motor command generation but also has applications in Brain Computer Interfaces (BCI). Traditionally, spectral power changes in the narrowband sensorimotor oscillations in EEG such as Event-Related (De)Synchronization (ERD/S) (Pfurtscheller and Lopes da Silva, 1999) are used to determine movement. Along with these narrowband spectral processes, changes also occur in the temporal processes in EEG. EEG shows Long-Range Temporal Correlation (LRTC) (Linkenkaer-Hansen et al., 2001; Hardstone et al., 2012) because of the power-law decay of its autocorrelation. The alpha band amplitude envelope shows LRTC, which decreases during movement (Linkenkaer-Hansen et al., 2004). Despite both the temporal and spectral changes in EEG, narrowband spectral features such as ERD are explored more commonly (Yuan and He, 2014; He et al., 2015), especially for movement detection and its applications in BCI. The LRTC during movement is also primarily obtained on the narrowband alpha amplitude envelope of the longer segments of EEG (Linkenkaer-Hansen et al., 2001; Zhigalov et al., 2016). Such LRTCs require a repeated movement and cannot be used for detecting single movement in real-time from the ongoing EEG. There is a paucity of research studying the ongoing temporal changes in the broadband EEG to detect movement on the single trial basis. In our previous study, we found that the autocorrelation of the broadband EEG decayed slower during movement (Wairagkar et al., 2018). We, therefore, investigate the temporal dependencies in the broadband EEG here in detail and also study their relationship with the ERD.

The temporal dynamics in the brain processes can be assessed by studying the temporal dependencies in EEG. These temporal dependencies can be directly observed from the autocorrelation function (ACF) of EEG. If the autocorrelation becomes zero after finite time lags or decays exponentially, then the time series exhibits a Short-Range Dependence (SRD), otherwise, if the autocorrelation decays slower than the exponential, then it has a Long-Range Dependence (Torre et al., 2007; Dette et al., 2017). A specific case of a long-range dependence is an LRTC which is characterized by the power-law decay of the autocorrelation function (Botcharova, 2014). LRTCs are widely observed in neuronal processes recorded at different levels that show power-law scaling such as neuronal firings (Hu et al., 2013), neuronal avalanches (Benayoun et al., 2010; Palva et al., 2013), local field potentials (Benayoun et al., 2010), electrocorticography, and non-invasive EEG and magnetoencephalography (Nikulin and Brismar, 2005; Benayoun et al., 2010). The spontaneous EEG spectrum is of the form 1/*f*, which shows power-law scaling and LRTC (Nikulin and Brismar, 2005; Berthouze et al., 2010). LRTCs have been ubiquitously observed in both oscillatory and non-oscillatory neuronal processes in EEG such as various narrowband oscillation amplitude envelope fluctuations (Linkenkaer-Hansen et al., 2001; Hardstone et al., 2012), phase of alpha oscillation (Botcharova, 2014), broadband phase synchrony (Kitzbichler et al., 2009), avalanches (Benayoun et al., 2010; Palva et al., 2013), and the energy profile of EEG (Parish et al., 2004; Benayoun et al., 2010).

The LRTC in EEG is typically obtained in the alpha band amplitude envelope fluctuations (Linkenkaer-Hansen et al., 2004; Zhigalov et al., 2016). During movement, the LRTC in the alpha amplitude fluctuations decreases, possibly due to the disruption caused in the long-memory process by movement (Linkenkaer-Hansen et al., 2004; Botcharova et al., 2014, 2015). However, these alpha amplitude LRTCs completely disregard the LRTCs in the broadband EEG. The brain rhythms are non-stationary and not strictly restricted to the selected narrow sinusoidal frequency bands (Cole and Voytek, 2018), and hence the LRTCs computed on such bands can overlook important features present in the entire power spectrum. The arrhythmic broadband processes and oscillatory processes coexist in neuronal activity (He, 2014). The arrhythmic broadband brain activity was previously considered background noise; however, recent studies demonstrate that it is physiologically and functionally relevant (He, 2014). The dynamics of arrhythmic broadband EEG change with task demands and cognitive states, and it has been associated with the excitation/inhibition balance of neuronal populations (Haller et al., 2018). It is interesting to determine the unexplored changes in such temporal dynamics of the arrhythmic broadband EEG during voluntary movement. The traditional narrowband alpha amplitude fluctuation LRTC requires long EEG segments (Linkenkaer-Hansen et al., 2001), and LRTC is considered an invariant property of brain dynamics over several scales. This approach does not facilitate observation of the ongoing instantaneous changes in LRTC. Berthouze and Farmer (2012) characterized changes in LRTC using a Kalman filter, but their timescales were several seconds long. Here we focus on shorter timescales by computing instantaneous changes in the LRTC using 2 s broadband EEG sliding windows to detect movement intention as opposed to a single LRTC measure for the entire duration of EEG. We call these continuous, instantaneous LRTC measures on short sliding EEG windows as ongoing LRTC. This allows us to observe the real-time changes occurring in the broadband LRTC, which is not possible with the traditional approach.

The LRTC can be quantified by the exponent of the power-law decay (α) of the ACF (ρ(*t*)) following the relation ρ(*t*) = *Ct*^−α^, where *C* is a constant. Equivalently, LRTC can also be quantified by the exponent (β) of the power spectrum of EEG (*S*(*f*)) obtained by taking the Fourier transform of ACF which follows the relation *S*(*f*) = *Bf*^−β^, where *B* is a constant. This gives an equality β = 1 − α (Botcharova, 2014). More conveniently, the Hurst exponent (*H*) can be used to quantify LRTC reliably because using the above methods is challenging in practice (Rangarajan and Ding, 2000; Delignieres et al., 2006). The relation between *H* and β is $H=\frac{1-\beta }{2}$ (Rangarajan and Ding, 2000). Detrended Fluctuation Analysis (DFA) (Peng et al., 1995) is commonly used to estimate the Hurst exponent because it is effective on a non-stationary time series (Kantelhardt et al., 2001; Delignieres et al., 2006; Hardstone et al., 2012).

In this study, we not only identify the LRTCs in the ongoing dynamics of the broadband EEG during movement but also model them. One of the models for modeling the long-range dependency is the Autoregressive Fractionally Integrated Moving Average (ARFIMA) model. ARFIMA(*p, d, q*) contains three components: the Autoregressive (AR) process of order *p*, the Moving Average (MA) process of order *q*, and the long-range dependence parameter *d* (*d* = *H*−0.5) (Wagenmakers et al., 2004). ARFIMA is a generalization of the Autoregressive Integrated Moving Average (ARIMA) model which is used to model a non-stationary time series. An integrated model such as ARIMA(*p, d, q*) makes the time series stationary by differencing it *d* times, after which the remaining stationary parameters AR and MA can be estimated (Box and Jenkins, 2015). In the case of time series such as EEG which has long-range dependencies and 1/*f* power spectrum, the time series must be fractionally differenced to make it stationary. Hence the ARFIMA(*p, d, q*) model with a fractional differencing parameter *d* is more suitable for EEG (Wagenmakers et al., 2004). We chose the ARFIMA model because it allows modeling both short- and long-range dependencies simultaneously, which enabled us to investigate the changes in the ongoing dynamics of both the types of dependencies during voluntary movement intention and execution. ARFIMA is useful for modeling broadband EEG because these types of parametric models can describe the second-order statistics of time series completely (Schlögl et al., 2000). To our knowledge, ongoing changes in the dynamics of LRTC and SRD in the broadband EEG on short windows were not investigated during motor command generation and used for detection of movement on a single trial basis.

In the literature, parametric models for time series analysis such as autoregressive, adaptive autoregressive, multivariate adaptive autoregressive (Hettiarachchi et al., 2015) models were used for movement detection from EEG. However, these attempted to model only selected frequency band amplitudes such as alpha and beta. None of the studies modeled the broadband EEG along with its short- and long-range components.

We also investigated the relationship between these temporal dependencies in the broadband EEG with ERD during movement, which remains unexplored in the literature. We hypothesize that the ARFIMA parameters related to broadband LRTC and SRD can provide additional information about the movement which is complementary to the commonly used narrowband ERD and provide deeper insights into the processes involved in the motor command generation. We hypothesize that these new neural correlates introduced in this paper can also be used in motor-based BCIs where it is important to detect movement in real-time and identify changes in EEG associated with movement on short timescales. Hence, we focus on the 2 s sliding window to estimate ARFIMA features on a single trial basis to detect movement intention with high accuracies and explore the possibility of using these features for BCI.

The focus of this paper is to investigate the fundamental changes in the temporal dynamics of EEG during movement intention from all the available information present in broadband EEG without restricting to a particular narrowband. Second, to test whether these new broadband characteristics can be used to detect movement intention on single trials for potential applications in BCI. Thus, in this paper, we aim (1) to provide a complete characterization of temporal dependencies (SRD/LRTC) in broadband EEG during movement; (2) to accomplish this using the ARFIMA model and study the effect of movement on its parameters; (3) to investigate the complementarity of narrowband ERD and the broadband temporal dependencies LRTC and SRD; (4) to use the three independent streams of information provided by LRTC, SRD, and ERD to classify movement intention for applications in BCI.

## 2. Materials and Methods

### 2.1. Participants

EEG was recorded from 14 healthy participants (8 female, age 26 ± 4 years, 12 right-handed) with no prior EEG and BCI experience. This study was carried out following the recommendations of the human experimentation guidelines of the University of Reading. The ethical approval for the EEG experiment was obtained from the ethics committee of the School of Systems Engineering, University of Reading, UK. Informed written consent was obtained from all participants before the EEG recording in accordance with the Declaration of Helsinki.

### 2.2. Experimental Paradigm

We chose a self-paced, asynchronous single index finger tapping task for EEG recording. Each EEG trial started with a fixation cross at the center of the screen for 2 s followed by an instruction for a single right index finger tap, left index finger tap and resting state in random order. Our aim was to investigate changes in broadband EEG related to right- and left-hand movement intention. The participants were asked to perform the task at any random time of their choice within a window of 10 s following the instruction. To avoid cue effects, we instructed participants not to tap immediately after the display of the instruction. There was a break of 1–1.5 s at the end of each trial. The experimental paradigm was developed in MATLAB Simulink R2014a (The MathWorks, Inc., Natick, Massachusetts, United States) using the BioSig toolbox (Vidaurre et al., 2011).

We recorded 40 EEG trials per condition at the sampling rate of 1,024 Hz. EEG was later downsampled to 128 Hz for further processing. The impedances of all the electrodes were kept below 7 *k*Ω. We recorded EEG using a Deymed TruScan 32 EEG amplifier (Deymed Diagnostic s.r.o., Hronov, Czech Republic) and EASYCAP EEG cap (EASYCAP GmbH, Herrsching, Germany). In this study, we used channels C3, Cz, and C4 over the motor cortex according to the international 10–20 system out of the 19 EEG channels recorded with a referential montage with reference on FCz and ground on AFz to study movement-related changes in EEG.

We recorded the onset of a finger tap with a bespoke microcontroller tapping device developed using an 8-bit Microchip PICDEM2 Plus demo board (Microchip Technology Inc., Arizona, USA) at 1,000 Hz. The participants placed both the index fingers in the corresponding finger caps of the tapping device. The two channels of binary tapping signals, capturing the onset and duration of each finger tap were co-registered with EEG using TOBI SignalServer 2.0 protocol (Breitwieser et al., 2012). EEG data is available from http://dx.doi.org/10.17864/1947.117 (Wairagkar, 2017). The details of the experimental paradigm and artifact removal are given in (Wairagkar et al., 2018).

#### 2.2.1. Pre-processing and Artifacts Removal

We performed all EEG analyses offline in MATLAB. EEG was filtered using a fourth-order zero-phase non-causal Butterworth filter to avoid phase distortions. The power-line noise was removed with a notch filter at 50 Hz. We removed the DC offset and the high-frequency noise from EEG by band-pass filtering between 0.5 and 45 Hz.

We performed artifact removal using independent component analysis (ICA) (Jung et al., 2000) with EEGLAB toolbox for MATLAB (Delorme and Makeig, 2004), which uses an automated version of infomax ICA (Makeig et al., 1997). We manually identified and removed the independent components with artifacts. We visually inspected the reconstructed uncontaminated EEG again and eliminated any undesirable trials containing large residual artifacts. EEG was segmented into time-locked trials of 6 s (−3 s to +3 s from the onset of the finger tap). These trials were divided into 2 s sliding windows from time *t* − 2 s to *t* shifted by 100 ms. The instantaneous indices obtained at time *t* for a single trial were computed independently on a 2 s broadband (0.5–45 Hz) EEG causal window from *t*−2 s to *t*. Thus, we computed new features every 100 ms to avoid causing a lag in BCI. We chose window length of 2 s because it provided a sufficient number of timescales to robustly compute the Hurst exponent using DFA analysis as detailed in the next section without compromising its instantaneous characterization which was essential for studying ongoing temporal dynamics.

### 2.3. Identifying Long-Range Temporal Correlation (LRTC) in Broadband EEG Using Detrended Fluctuation Analysis (DFA)

Whether a time series has a long- or short-range dependence can be identified from the decay of its autocorrelation function (ACF) and log-log plot of its power spectrum. If the ACF decays according to the power-law, and the log-log power spectrum shows a straight line, then the time series has LRTC. If the ACF decays exponentially, and the log-log power spectrum is not linear, then it has SRD (Wagenmakers et al., 2004). To identify the temporal dependencies in the 2 s broadband EEG, we computed the ACF and log-log power spectrum obtained by squaring the Fourier transform. We plotted the grand average ACF and power spectrum of all the windows in all the trials in all the participants. The LRTCs can be quantified by Hurst exponent using DFA (Peng et al., 1995) in a non-stationary time series such as EEG by avoiding the artifactual dependencies caused by non-stationarity and trends (Peng et al., 1995; Kantelhardt et al., 2001). We performed the DFA on each 2 s sliding window of the broadband EEG which gave an estimate of the Hurst exponent *H* every 100 ms. The Hanning window was applied to each 2 s EEG segment before the DFA to avoid edge effects. The DFA was performed as follows:

1. The 2 s EEG window *X* of length *N* (256 samples) was integrated according to Equation (1).
  (1) $$\begin{array}{r} Y_{k}=\overset{k}{\underset{i=1}{\sum }}X_{i}-\bar{X} \end{array}$$
  where, *k* = 1, …, *N* and *Y* is the integrated time series. $\bar{X}$ is the mean of *X*.
2. The integrated time series *Y* was divided into *N*/*n* non-overlapping boxes of length *n*, where *n* is an individual timescale at which we computed the Root Mean Square (RMS) fluctuations. We chose the timescales of *n* = [10, *N*/4] samples (i.e., [78 ms, 0.5 s]). The box sizes of *n* = [10, *N*/4] are commonly used to get good estimate of RMS fluctuations at each timescale (Delignieres et al., 2006; Botcharova et al., 2013). We used 25 box sizes between *n* = [10, *N*/4] equidistant on the *log*_2_ scale as our number of samples was a power of 2. Delignieres et al. (2006) have shown that we can obtain correct estimates of Hurst exponent using a short time series of length 256 with DFA. Our box sizes were within the range suggested by Li et al. (2018) $\left[max\left(k+2,\frac{F_{s}}{F_{max}}\right),min\left(\frac{N}{4},\frac{F_{s}}{F_{min}}\right)\right]$ where *k* = 1 (linear detrending in DFA) for filtered data between *F*_*min*_ (0.5 Hz) and *F*_*max*_ (45 Hz).
3. At each scale *n*, for every non-overlapping segment of *Y* of length *n*, a trend was obtained by the least square linear fit. *Y*_*n*_ is a concatenation of trends at a scale *n* for all the *N*/*n* boxes and the RMS fluctuations were computed according to Equation (2) for each *n* = [10, *N*/4].
  (2) $$F_{n}=\sqrt{\frac{1}{N}\sum_{i=1}^{N}{\left(Y_{i}-Y_{n,i}\right)}^{2}}$$
4. *N* was not fully divisible by *n* for each box size. Hence, we obtained the final RMS fluctuations by averaging the *F*_*n*_ computed using the steps 1–3 from the forward and backward direction of each EEG window *X* (Kantelhardt et al., 2001).
5. We computed the Hurst exponent by obtaining the slope of the linear fit to the log-log plot of RMS fluctuations at each timescale *n* (*log*_2_*F*(*n*) vs. *log*_2_*n*).

After obtaining *H* for each EEG window, an exponential smoothing filter was used to smooth *H* in the consecutive sliding windows in the single trials to avoid noisy estimates.

The Hurst exponent estimated using DFA is valid and suggests the presence of the power-law in the fluctuations at different timescales only if the log-log DFA plot is linear. We validated the Hurst exponents *H* by assessing the linearity of the log-log DFA plot by comparing the fit of the linear, polynomial, logarithmic and exponential models to it using the maximum likelihood-DFA (ML-DFA) method detailed in Botcharova et al. (2013). The LRTC is present in the time series if the Hurst exponent is between 0.5 and 1 (Kantelhardt et al., 2001; Linkenkaer-Hansen et al., 2001).

### 2.4. Modeling the Broadband EEG Using Autoregressive Fractionally Integrated Moving Average (ARFIMA) Model

AFRIMA allows simultaneous estimation of both LRTC and SRD in a time series (Wagenmakers et al., 2004). ARFIMA(*p*,*d*,*q*) incorporates SRD processes through the AR parameters *p* and the MA parameters *q*, and the LRTC process through the fractional differencing parameter *d*. The ARFIMA(*p*,*d*,*q*) model is given by Equation (3) (Botcharova, 2014).

(3) $$\begin{array}{r} \left(1-\overset{p}{\underset{i=1}{\sum }}\phi_{i}B^{i}\right){\left(1-B\right)}^{d}X_{t}=\left(1+\overset{q}{\underset{i=1}{\sum }}\theta_{i}B^{i}\right)\epsilon_{t} \end{array}$$

*B* is the backshift operator, such that *BX*_*t*_ = *X*_*t*−1_ and $B^{n}X_{t}=X_{t-n}$, ϕ_*p*_ are the AR coefficients of the order *p*, θ_*q*_ are the MA coefficients of the order *q* and ϵ_*t*_ is innovation at time *t* drawn from a normal distribution. For ARFIMA, *d* can have a fractional value. We estimated the ARFIMA(*p*,*d*,*q*) for each 2 s sliding window of the single trial broadband EEG by firstly fractionally differencing the series with *d* and then estimating the parameters of ARMA(*p*,*q*) as described in the following sections.

#### 2.4.1. Removing LRTC From EEG With Fractional Differencing

The parameter *d* accounts for the LRTC in the ARFIMA process. The AR and MA parameters can only be estimated accurately for a stationary SRD process. The first step of fitting ARFIMA was to fractionally difference each 2 s EEG window by its corresponding fractional differencing parameter *d* to remove LRTC and make it stationary. The parameter *d* = *H* − 0.5, where *H* was estimated by the DFA method described in the previous section 2.3. The fractional differencing can be performed using a binomial series expansion as given in the Equation (4) with a Gamma function (Granger and Joyeux, 1980; Baillie, 1996; Liu et al., 2017). We used the Matlab fast fractional difference algorithm provided in Jensen and Nielsen (2014) for fractionally differencing each EEG window.

(4) $$\begin{array}{r} {\left(1-B\right)}^{d}=\overset{\infty }{\underset{k=0}{\sum }}\left(\begin{array}{c} d\\ k \end{array}\right){\left(-B\right)}^{k} \end{array}$$

#### 2.4.2. Identification of the Order of the ARMA(*p,q*)

The AR and MA parameters *p* and *q* of the ARFIMA were estimated by fitting the ARMA(*p*,*q*) model to the fractionally differenced EEG window. The stability of the model was assessed by confirming the stationarity of the time series using the augmented Dicky-Fuller test for unit root (Im et al., 2003). We then identified the model order of ARMA for each 2 s EEG window using Akaike Information Criterion (AIC) (Akaike, 1974) by comparing the models with *p* ranging from 1 to 10 and *q* ranging from 1 to *p* − 1. The order of the best fitting ARMA(*p*,*q*) model which gave the least AIC for the maximum number of EEG windows in all the participants was chosen as the order of the AR and MA parameters of ARFIMA.

#### 2.4.3. Estimation of ARFIMA(*p,d,q*)

Having identified the order of the ARMA part of the ARFIMA, we then estimated the AR and MA parameters *p* and *q*, respectively, using the Matlab functions *arima()* and *estimate()* from Econometrics toolbox (The MathWorks Inc., 2018) for each 2 s sliding broadband EEG window. The residual analysis was performed on the estimated model using the Ljung-Box Q test (Ljung and Box, 1978) to assess whether the residuals have any significant autocorrelation and one-sample Kolmogorov–Smirnov test (Massey, 1951) to evaluate whether the residuals have a normal distribution. All the parameters *d*, *p*, and *q* obtained for each window were then plotted to assess the ongoing change during the trial.

### 2.5. Event-Related Desynchronization (ERD) on Single Trial EEG

The ERD analysis was also done on individual 2 s EEG sliding windows. We used the band power method for characterizing the ERD (Pfurtscheller and Lopes da Silva, 1999). Each EEG window was band-pass filtered between 8 and 13 Hz (alpha band). The mean of each window was subtracted from itself. The analytic signal obtained from the absolute value of the Hilbert transform of EEG was used to get the amplitude of the alpha band. The band power was computed by taking the mean of the squared alpha band amplitude. ERD is the relative change in the alpha band power from the baseline during movement. We computed the baseline alpha band power *R* for individual participants by averaging the band powers of all the 2 s windows in all the resting state trials. We then computed the percent ERD at each time *t* by subtracting the baseline *R* from the band power of 2 s sliding EEG window *A*_*t*_ using $ERD_{t}=\left(\frac{A_{t}-R}{R}\right)\times \;100$.

We also evaluated ERD as above on the fractionally differenced EEG windows to observe the effect of removal of LRTC from EEG on the ERD in the alpha band.

### 2.6. Hybrid Classifier for Movement Intention Detection

From the temporal and spectral EEG analysis described in the previous sections, we computed three types of features: broadband LRTC obtained from DFA, broadband SRD obtained from the parameters of ARFIMA and well-known narrowband ERD which could be used for detecting movement from EEG. We performed the classification using various combinations of the LRTC, SRD, and ERD features to identify right tap vs. resting state and left tap vs. resting state independently using binary Linear Discriminant Analysis (LDA). Our goal was to compare the performance of the classifier for movement intention detection using these features independently and with hybrid combinations of these features.

The classification was done on each participant independently. We trained a separate LDA classifier for each sliding window with the feature vectors from corresponding windows in all the movement trials and the same number of features vectors randomly selected from the resting state trials of that participant. Each LDA had 40 data samples (equivalent to the number of trials) with the selected number of features for each class. We used 10 × 10 fold cross-validation to assess the performance of the classifier by obtaining the classification accuracies, sensitivities and specificities at each time point in the trial given by 2 s sliding windows. The 95% confidence level for binary classification (movement vs. rest) was obtained from the binomial distribution with *n* = number of EEG trials and *p* = 0.05. We also noted the time at which classifier crossed this threshold as the time of significantly identifying the movement intention.

## 3. Results

### 3.1. Autocorrelation Function and Power Spectrum of Broadband EEG During Voluntary Movement

**Figure 1:**
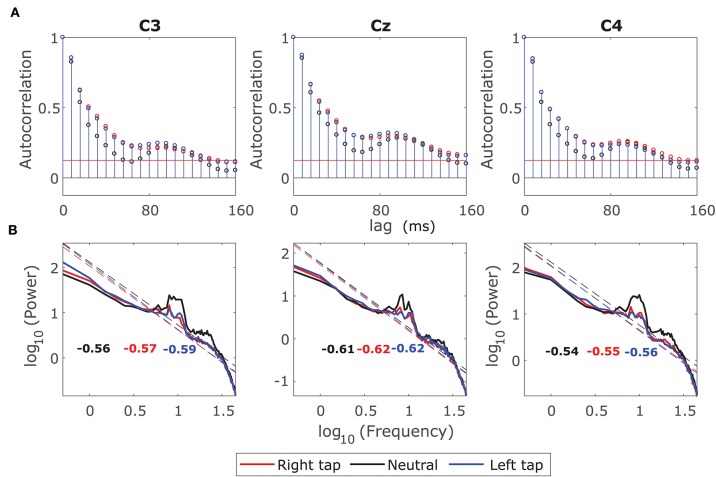
Grand average autocorrelation function (ACF) and power spectrum of EEG during movement. **(A)** The grand average ACF of 2 s broadband EEG from −1 s to +1 s for all the participants for right finger tapping (red), left finger tapping (blue), and resting state (black) in channels C3, Cz, and C4. The ACF decays slowly indicating the LRD. **(B)** The grand average log-log power spectrum in the channels C3, C4, and Cz for all the three conditions. The log-log power spectrum is linear in all the cases with slopes between −0.5 and −1.5 suggesting the presence of LRTC.

We plotted the ACF and the log-log power spectrum of a 2 s EEG window from −1 s to +1 s of movement onset such that it contained the information regarding movement intention and execution. We also plotted the ACF and the log-log power spectrum of the corresponding resting state EEG 2 s window. Figure 1A shows that the ACF decays slowly (slower than exponential) in all the conditions indicating the presence of long-range dependence. The ACF decays slower for right and left finger tapping, indicating an increase in long-range dependence. Figure 1B shows the log-log power spectrum, which is linear in all the three conditions with a peak at 10 Hz as expected. The linear log-log power spectrum suggests that the dependence is long-ranged. If the slope of the power spectrum is between −0.5 to −1.5, then it indicates LRTC in the time series (Wagenmakers et al., 2004). The slopes that we observe are in the valid range for LRTC with a slightly increased slope for right and left finger tapping. We explore the increase in LRTC during the voluntary movement further in the next sections because it is not practical to identify the exponent for LRTC robustly from either autocorrelation or power spectrum of a non-stationary process such as EEG, especially on single trials. Hence, we use DFA analysis in the next section to compute Hurst exponent robustly on single EEG windows. The 10 Hz peaks for right and left finger tapping have lower power than that of the resting state peak, especially in channels C3 and C4; this represents the ERD during voluntary movement.

### 3.2. LRTC in the Broadband EEG Using DFA

**Figure 2:**
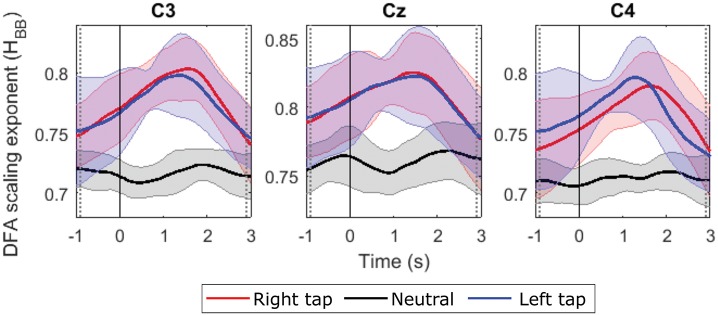
The grand average time evolution of Hurst exponents of broadband EEG (*H* quantifying LRTC). The progressions of grand average *H* in all the participants in channels C3, Cz, and C4 during right finger tap (red), left finger tap (blue), and resting state (black). The LRTC increases during movement intention and execution. The movement onset is at 0 s, marked by a solid vertical line. The *H* of movement trials is significantly different from the *H* of resting state trials in the time region between the dotted gray vertical lines (*p* < 0.05). The shaded areas show the standard deviation.

We obtained valid Hurst exponents for the LRTCs in 2 s broadband EEG windows in the range of 0.5 to 1 using DFA. The ML-DFA validated the DFA scaling exponents by selecting the linear model as the best fitting model to the log-log DFA fluctuation plots confirming the presence of the LRTCs in the broadband EEG. The LRTC was present in EEG in the movement state as well as in the resting state. Figure 2 shows the grand average Hurst exponent throughout EEG trial of all the participants during movement and resting states. We can see a clear increase in the LRTC during movement intention and execution. The Hurst exponent *H* increased significantly from the resting state between the vertical gray dotted bars (*p* < 0.05, Mann–Whitney *U*-test, *n* = 14; number of participants) approximately between −1 and 3 s of the onset of movement. The *H* was used to obtain the fractional differencing parameter *d* for ARFIMA by subtracting 0.5 from it. We did not observe any lateralization in the LRTC during right- and left-finger taps. Channels C3, Cz, and C4 showed maximum changes in the Hurst exponent. It would be interesting to study the localization of LRTC in the future using spatial filters.

### 3.3. Modeling the Broadband EEG Using ARFIMA(*p,d,q*)

#### 3.3.1. Removal of LRTC From EEG With Fractional Differencing

**Figure 3:**
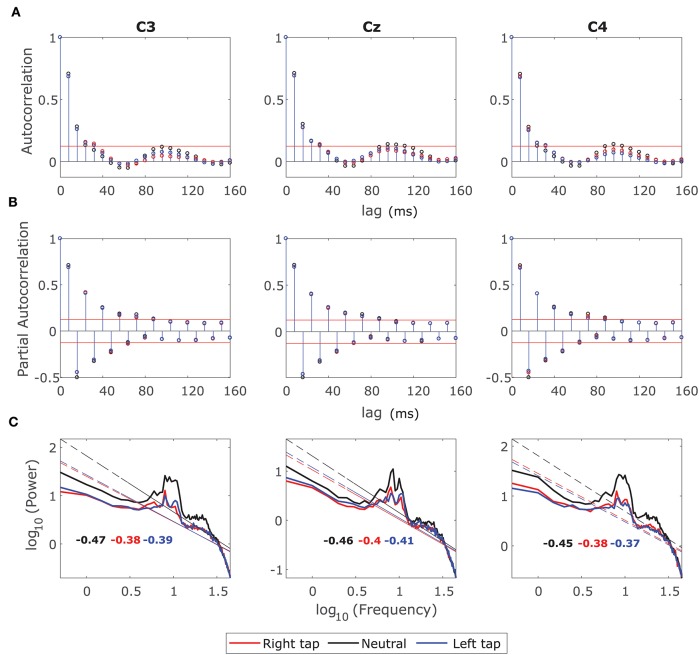
Grand average autocorrelation function (ACF), partial autocorrelation function (PACF), and power spectrum of the fractionally differenced EEG. **(A)** The grand average ACF of 2 s broadband fractionally differenced EEG from −1 s to +1 s for all the participants for right finger tapping (red), left finger tapping (blue), and resting state (black) in channels C3, Cz, and C4. The ACF decays fast indicating SRD. **(B)** The grand average of PACF in all the three channels in movement and resting state conditions, which cuts off after nine lags. **(C)** The grand average log-log power spectrum in the channels C3, C4, and Cz for all the three conditions. The log-log power spectrum is no more linear with slopes outside the range of LRTC (−0.5 and −1.5).

We examined the ACF and the log-log power spectrum of the fractionally differenced EEG windows with *d* to identify whether the LRTC has been removed so that the ARMA(*p, q*) model could be fitted. Figure 3 shows the grand average ACF, Partial Autocorrelation Function (PACF) and log-log power spectrum of the fractionally differenced 2 s broadband EEG windows from −1 s to +1 s of movement onset in all the three conditions. Figure 3A shows that the ACF decays fast (after four lags) after fractional differencing and Figure 3B shows that the PACF cuts off after nine lags suggesting the presence of the SRD in the residual EEG.

The log-log power spectrum is also no longer linear. Figure 3C shows that the lower frequencies have been flattened creating a bend in the power spectrum, which again confirms the removal of LRTC. The slopes of the power spectra are also outside the range of −0.5 and −1.5 for the LRTC. The decrease in alpha power in the right and left movement conditions is still visible in the channels C3 and C4 as smaller 10 Hz peaks than that of the resting state, which is consistent with the ERD.

#### 3.3.2. Identification of the Order of the ARMA(*p,q*) Model

The fractionally differenced EEG was confirmed to be stationary by the augmented Dicky-Fuller test for unit roots (*p* < 0.05). EEG windows did not have unit roots indicating the stability of the ARMA model to be fitted to the short-range dependent process.

Figure 3B shows that the PACF cuts off after nine lags, which can suggest the initial estimate of the AR order could be 9. According to the AIC, the order of the best fitting model was ARMA(10,0) for 85% of times of all the windows in all the trials in all the channels and all the participants. In some cases where the order of MA (*q*) was greater than zero, the roots were non-invertible, and hence we set *q* = 0 since ARMA(*p, q*) can also be represented by AR(*p*) with higher order. The distribution of the selected orders of the models by AIC remained the same for all channels, all participants, all conditions before movement, during movement and after the movement. Hence we selected ARMA(10,0) for modeling the SRD in fractionally differenced EEG.

#### 3.3.3. Estimation of ARFIMA(*10,d,0*)

The AR parameters of the order 10 were estimated, and the residual analysis was performed on the residue of the model ARMA(10,0). The residual analysis using the Ljung-Box Q test showed that for 95% of all EEG windows, the estimated ARMA(10,0) fitted well (*p* < 0.05) to the SRD process in the fractionally differenced EEG and there was no more information in the residuals left to be modeled. The Kolmogorov–Smirnov test confirmed that the residuals of all EEG windows had a normal distribution (*p* < 0.05). Thus, we modeled 2 s broadband EEG windows successfully using ARFIMA(10,*d*,0) and estimated the eleven model parameters (*d* and ten parameters for AR).

### 3.4. Changes in the Long-Range and Short-Range Temporal Dependence Identified From the Ongoing ARFIMA(10,*d*,0) During Movement

**Figure 4:**
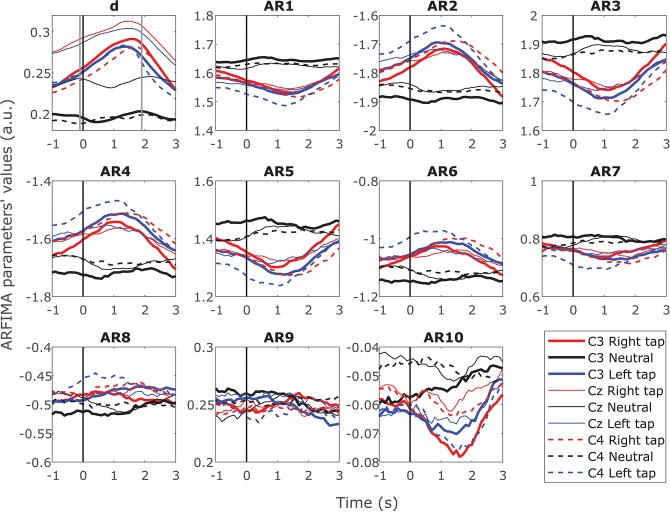
Time progression of the grand average ARFIMA(10,*d*,0) estimated parameters for the broadband EEG. The time progression of 10 grand average AR parameters reflecting the dynamics of short-range dependence and fractional differencing parameter *d* reflecting the dynamics of long-range dependence (LRTC) throughout the trial in C3, Cz, and C4 for right finger tapping, left finger tapping and resting state. The movement onset is at 0 s marked by a solid vertical line. There were significant changes in the AR parameters for SRD during the movement for 10 individual participants. However, the grand average AR parameters did not show significant change due to inter-participant variability. The parameter *d* increased significantly (*p* < 0.05) during the movement.

ARFIMA incorporated the LRTC through the parameter *d* and the SRD through ten AR parameters. Figure 4 shows the grand average time progressions of these ARFIMA parameters throughout the movement trial in all the participants. A clear increase in the parameter *d* was observed during movement intention and execution. The parameter *d* was significantly different in movement trials and resting state trials (*p* < 0.05, Mann–Whitney *U*-test, *n* = 203688; individual windows on which parameters were estimated in all the participants). The first six of the ten AR parameters showed change during movement in all the three channels. Though there is a change during movement, there is no significant difference in the individual parameter in the movement trials vs. resting state trial for all the participants together. Thus, on the grand average, during movement, there was a change in the SRD in broadband EEG though not significant, whereas, the LRTC in the broadband EEG increased significantly.

The same results were obtained on individual participants for *d* on single trials. However, there was larger variability in the AR parameters in the individual participants. Ten out of fourteen participants showed a significant change in the AR parameters between resting state trials and movement trials (*p* < 0.05, Mann–Whitney *U*-test, *n* = 40; number of trials, note that the *p*-value was obtained at each time point during the trial). Out of the 10 participants that showed a significant change, 80% showed a change in all the three channels and 90% showed a significant change in at least the first six AR parameters. These parameters had a higher magnitude. None of the participants showed a significant change in AR parameters 8, 9, and 10, which had a lower magnitude. The absolute values of the AR parameters decreased gradually in the higher order parameters. The consecutive order AR parameter values alternated between positive and negative, as seen in Figure 4. Even though ten individual participants show significant differences in their SRD dynamics, averaging over population in obscuring the information in individuals due to larger population variability than individual variability.

### 3.5. Complementarity of Long-Range Temporal Correlation and ERD

We established the complementarity of narrowband ERD and broadband LRTC by examining the effect of LRTC on estimating ERD by removing LRTC and by examining the effect of ERD on estimating LRTC by removing the ERD from EEG.

**Figure 5:**
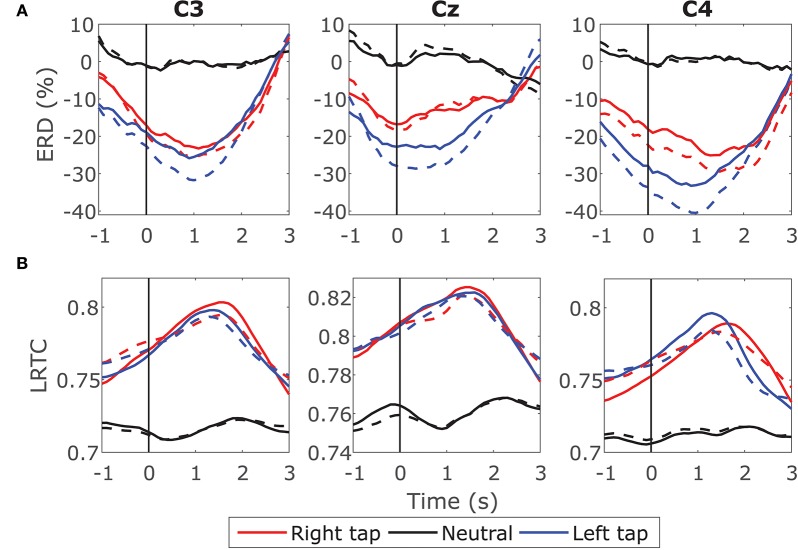
Effect of LRTC on ERD and effect of ERD on LRTC. **(A)** Solid lines show the time progression of grand average ERD calculated from EEG containing LRTCs in the right finger tap (red), left finger tap (blue), and resting state (black) trials from all the participants. The dotted lines represent the ERD after removing LRTCs from the broadband EEG by fractionally differencing it by *d*. The movement onset is at 0 s shown by a solid vertical line. Both the ERDs during movement are significantly different from the resting state (*p* < 0.05). ERDs with and without LRTCs are not significantly different. **(B)** Solid lines show the time progression of grand average LRTC calculated from EEG containing ERD in right tap, left tap and resting state. The dotted lines represent the LRTC after removing ERD from the broadband EEG by fixing the 8–13 Hz power. Both the LRTCs during movement are significantly different from the resting state (*p* < 0.05). LRTCs with and without ERD are not significantly different from each other. LRTC and ERD do not have a significant effect on each other because removing one does not change the values of the other.

First, we compared the ERD on raw EEG having LRTC and EEG after removing the LRTC using fractional differencing. Figure 5A shows the ERD in EEG with LRTC (solid line) and ERD in the same EEG after removing LRTC (dashed line) in right finger tap, left finger tap and resting state. Both the ERDs follow similar traces and show a significant decrease in the alpha band power during movement as compared to the resting state (*p* < 0.5, Mann–Whitney *U*-test, *n* = 14; number of participants) as expected. The difference between ERD when LRTC was present and ERD when the LRTC was absent was not significant in all the three conditions. Same results were obtained for the individual participants' ERD on single trials. Thus, the ERD is unaffected by the presence of LRTC in EEG.

We then tested the effect of ERD on LRTC by comparing the LRTC on raw EEG and LRTC on EEG with ERD removed. We suppressed the changes in ERD by fixing the magnitude of alpha power in all the windows of a trial. This was done by replacing the magnitude of 8–13 Hz in the Fourier spectrum of all the 2 s windows in a trial with the magnitude of 8–13 Hz from a randomly selected window from that trial and then reconstructing EEG time series by inverse Fourier transform. This ensured that the alpha power did not change throughout the trial resulting in a constant value of ERD at all the time points in a trial. Figure 5B shows the LRTC in EEG with ERD (solid line) and LRTC in EEG after suppressing the ERD (dashed line). There is no significant change in the two LRTCs. Thus, the LRTC is unaffected by ERD. This indicates that the broadband LRTC and narrowband ERD are distinct and complementary processes. Knowledge of ERD does not inform us about the values of LRTC and vice versa.

**Figure 6:**
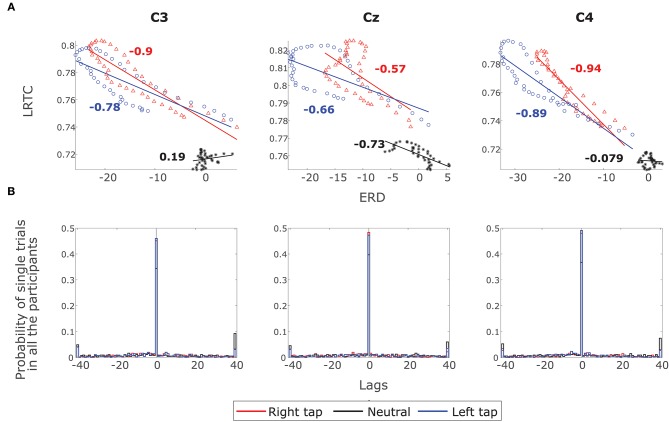
The correlation between ERD and LRTC. **(A)** Scatter plot shows a strong inverse correlation between ERD and LRTC during right (red) and left (blue) finger tapping in channels C3, Cz, and C4. There is no correlation between ERD and LRTC during resting state (black). The correlation coefficients are shown beside the fitted straight lines. **(B)** The distribution of lags with maximum cross-correlation between LRTC and ERD in single trials of all the participants in all the three conditions. The LRTC and ERD have maximum correlation at lag 0.

The ERD decreased, whereas the LRTC increased during the movement. The scatter plot of the grand average ERD and LRTC in Figure 6A shows a strong inverse correlation between them with the high correlation coefficients for right and left finger tap conditions. There was no correlation between ERD and LRTC during the resting state. During movement, there is a switch in the dynamics of ERD and LRTC, and they become coupled from their uncorrelated state during the resting condition.

Figure 6B shows the distribution of lags at which there is a maximum correlation between LRTC and ERD in single trials in all the participants in all the three conditions. The correlation is maximum at lag 0, which indicates that LRTC or ERD processes do not precede one another and occur at the same time.

### 3.6. Hybrid Classifier for Movement Intention Detection

We compared the classification accuracies for movement intention detection using hybrid features of LRTC, ARFIMA and ERD. For the LRTC features, we used *H* from all the three channels C3, Cz and C4 as LRTCs were observed in all the three channels with equal strength (Figure 2). For the ERD features, we used channel C3 and C4 as Cz showed relatively less distinction between movement and rest ERD (Figure 5). For the ARFIMA features, we used parameters *d* and AR1 to AR6 from all the three channels since these AR parameters significantly changed in most participants during movement (*p* < 0.05) leading to 21 features. The average classification accuracies, sensitivities, and specificities of all the participants using all the classifiers are shown in Table 1. The classification accuracies of individual participants are included in Supplementary Tables 1A,B.

**Table 1:** The average of peak LDA classification accuracies for the right and left finger movement vs. resting state of all the participants using LRTC, ARFIMA, ERD, hybrid LRTC+ERD and hybrid ARFIMA+ERD features.

		**Left finger tapping**			**Right finger tapping**		
		**Accuracy**	**Sensitivity**	**Specificity**	**Accuracy**	**Sensitivity**	**Specificity**
		**(%)**	**(%)**	**(%)**	**(%)**	**(%)**	**(%)**
LRTC	Mean	75.69	75.85	75.53	76.07	76.39	75.75
	SD	6.77	9.10	6.32	6.40	6.05	7.92
ARFIMA	Mean	88.05	87.35	88.75	86.54	86.92	86.15
	SD	4.75	5.51	4.53	4.98	5.20	5.77
ERD	Mean	72.98	83.00	62.95	71.04	79.78	62.30^*^
	SD	5.23	12.12	10.08	6.40	14.30	9.00
LRTC	Mean	77.82	78.50	77.13	78.62	78.37	78.86
+ ERD	SD	6.97	9.66	7.17	7.14	8.11	7.94
ARFIMA	Mean	89.04	89.29	88.79	87.58	87.56	87.60
+ ERD	SD	3.74	4.57	4.35	4.56	5.40	4.64

All values except the ones marked by

* *are significantly above chance level (p <0.05)*.

The LRTC features showed higher classification accuracies than the ERD features. There was no significant increase in the classification accuracies of the hybrid LRTC and ERD classifier and was similar to the LRTC classifier. Combining the LRTC and ERD features improved the classification accuracies marginally. The classifier with ARFIMA features (which contained features representing both short- and long-range dependence) gave significantly higher accuracies of 87.30±4.87% than LRTC and ERD and their combination (*p* < 10^−5^, Wilcoxon signed-rank test, *n* = 28; right and left hand accuracies of 14 participants). The hybrid classifier with ARFIMA and ERD gave the highest mean classification accuracy of 88.71±4.12% which was only marginally higher (by 1.4%) than ARFIMA. The ERD, LRTC and ARFIMA accuracies were also correlated, i.e., the participant with lower classification accuracy with LRTC and ARFIMA also showed lower classification accuracies with ERD.

The LRTC classification accuracies, sensitivities and specificities were all similar, which shows that this classifier is robust and reliable. The hybrid classifier of LRTC and ERD and ARFIMA and ERD also showed the same robustness. Interestingly, the ERD classifier had high sensitivity but low specificity leading to lower accuracy. This indicates that by using ERD, we are likely to identify movement with higher accuracy but also get more false positives during the resting state. Considering these results, including the LRTC and SRD temporal dependency features improves the movement intention detection instead of using only ERD. The LRTC and SRD provide complementary information to ERD about motor command generation.

#### 3.6.1. Timing of Movement Intention Detection

**Figure 7:**
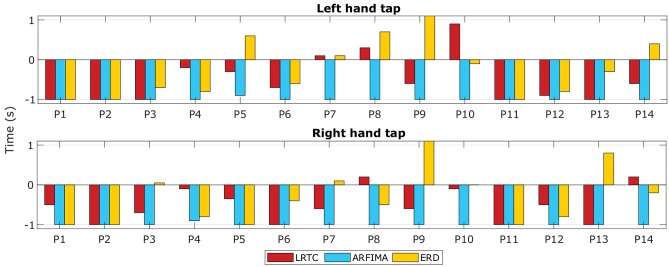
The timings of movement intention detection. The timings of movement intention detection when the classification accuracy crossed the significance threshold for right finger tap and left finger tap are shown for all the 14 participants. Timings obtained from classifiers using LRTC, ARFIMA, and ERD features are shown.

Figure 7 shows the time at which the classification accuracies crossed the significance threshold (chance level) in three types of classifiers. The LRTC, ERD and ARFIMA all were able to detect movement intention before its onset in most of the participants. On average, ERD detected movement −0.25 s of the actual movement onset, LRTC detected it at −0.5 s, and ARFIMA detected movement earliest at −1 s. Supplementary Figure 1 shows timings of all hybrid classifiers. All temporal dependency features detected movement earlier than ERD, which shows their suitability for application in BCI.

## 4. Discussion

We completely characterized the broadband temporal dependencies in EEG during movement by estimating the short and long-range temporal correlations. We have not only identified the ongoing changes in the dynamics of these temporal dependencies in the broadband EEG but also modeled them successfully using ARFIMA(10,*d*,0). Several other studies model EEG with ARMA models (Pfurtscheller et al., 2006; Hu, 2009; Chae et al., 2012; Resalat and Saba, 2016; Antelis et al., 2017), however, these models are suitable for processes with short-range dependencies. We have shown that the broadband EEG contains LRTCs and hence the ARFIMA model is more suitable, which incorporates both LRTC and SRD simultaneously. Monitoring the ongoing changes in both the LRTC and SRD continuously has resulted in the detection of movement intention with high accuracy, which has not been done before.

The LRTC increased significantly during the movement (*p* < 0.5) consistently in all the participants indicating that it is a robust correlate of movement. The SRD parameters also changed significantly (*p* < 0.5) in 71.4% participants, but there was a higher variability among participants. ARFIMA is a detailed mathematical model for time series, but it does not explain the underlying physiological processes and interpretation of EEG (Wagenmakers et al., 2004). The order of AR parameters of ARFIMA that we identified using AIC is in a similar range as the orders used by studies modeling EEG with AR which were between 9 and 13 by Florian and Pfurtscheller (1995), 10 by Fabiani et al. (2004), 8.67 by Tseng et al. (1995) and 16 by McFarland and Wolpaw (2008).

Most of the studies use AR and MA models for merely extracting the features from EEG, mostly from sensorimotor rhythms for BCI classification; however, these studies do not describe the nature of the ongoing changes in the model parameters during motor activity (Pfurtscheller et al., 2006; Hu, 2009; Chae et al., 2012; Resalat and Saba, 2016). The parametric ARMA models in the literature were used to characterize spectral features of specific narrowbands, especially of the sensorimotor rhythms for movement (Pfurtscheller et al., 2000; Fabiani et al., 2004; Chae et al., 2012; McFarland et al., 2015; Antelis et al., 2017). The ongoing changes in the broadband LRTCs were not studied previously and used for identification of movement intention. The broadband LRTC might even have implications in self-organized criticality in the brain (Linkenkaer-Hansen et al., 2001). LRTCs are observed in several brain processes (Botcharova et al., 2014). In EEG, LRTCs are typically observed in the envelope of alpha oscillations (Linkenkaer-Hansen et al., 2001). LRTCs are typically attributed to the theory of self-organized criticality in the brain facilitating the neural networks to quickly reorganized during varying processing demands (Linkenkaer-Hansen et al., 2001). However, further work will be required to study the mechanisms of broadband LRTC, and their relationship with self-organized criticality remains to be seen. These broadband temporal dynamics are more robust than ERD because they do not require selection of participant-specific frequency bands for better performance (Pfurtscheller and Lopes da Silva, 1999; Durka et al., 2001).

We discovered that the arrhythmic broadband LRTC and the rhythmic narrowband ERD are complementary processes and provide different information about movement intention. Removing the broadband LRTC from EEG did not affect the ERD and removing ERD from EEG did not affect LRTC significantly (Figure 5). The LRTC represented by broadband 1/*f* process and ERD represented by the alpha peak that resides over this 1/*f* spectrum are complementary movement-related neuronal process. Becker et al. (2018) suggested that the alpha power causes the change in LRTC in lower frequency band by observing a negative lag of maximum correlation between alpha power and LRTC in the spontaneous EEG. However, negative lag is not sufficient to conclude the causal effect between the alpha power and LRTC. Our results show that the broadband LRTC and ERD have inverse correlation during movement and the maximum correlation magnitude was at lag zero (see Figure 6), and thus both occur at the same time. The co-evolution of ERD and broadband LRTC may be because of a common input that drives both the processes during motor command generation.

Another independent neuronal process known to occur during movement intention is Motor-Related Cortical Potential (MRCP) or Bereitschafts potential (Shibasaki and Hallett, 2006; Bai et al., 2011). Burke et al. (2005) used an AR model with Bereitschafts potential as an exogenous input for movement detection and observed increased classification accuracy by combining the features from complementary processes. We have previously shown that the changes in the temporal dependencies are also complementary to the MRCPs (Wairagkar et al., 2015) because MRCP which is commonly observed in low frequencies (< 2 Hz) characterizes slow trend in EEG by eliminating the fluctuations, and on the contrary, the broadband LRTC characterizes the dynamics in the fluctuations by eliminating the slow trends using DFA. Thus, we have independent processes of LRTC, ERD, and MRCP containing complementary information about motor command generation.

We have confirmed that the LRTCs can be detected on short windows in broadband with rigorous analysis and ML-DFA (Botcharova et al., 2013) for validating the Hurst exponent. ARFIMA showed that the broadband EEG contained both short- and long-range temporal correlations. We obtained new model parameters every 100 ms describing the ongoing changes in the LRTC and SRD using an online type of processing pipeline that allowed us to detect the movement intention on single trials. The sliding window approach for model parameter estimation has been commonly used for non-stationary time series (Antelis et al., 2017). However, the model estimation is computationally expensive and will need efficient execution for BCI applications. The LRTC estimation, on the other hand, is achievable within the available time window for online BCI applications.

The hybrid classifier with ARFIMA parameters and ERD has a feature vector with high dimensionality and gives high classification accuracies. It was clear that increasing the feature vector dimensionality improved the classification accuracy significantly (*p* < 10^−5^) than just ERD, LRTC or their combination, which had a maximum of five features. We used 10 × 10 fold cross-validation scheme, and hence, the improved accuracy was not due to overfitting. The ARFIMA parameters reinforce the difference between the resting state and movement as multiple parameters show a clear distinction between the two conditions. Higher dimensionality made the feature space sparse, making it easier for LDA to find an optimum classification boundary. It is common in BCI to have feature vectors of high dimensionality, as generally several features are extracted from several channels and used for classification (Lotte et al., 2007). For example, the study in Hettiarachchi et al. (2015) used 52 features with 70 EEG trials per class. The training sets in BCI are often relatively small because of the time-consuming EEG recording processes, which is also exhausting for the participants (Lotte et al., 2007). Our hybrid classifier thus follows standard practices in BCI, giving high classification accuracies. However, it would be interesting to observe whether the classification accuracies using ARFIMA features remain the same after increasing the number of trials significantly. It would be interesting to investigate in future whether these LRTCs provide more information about the movement such as its kinematics. Based on our previous study (Wairagkar et al., 2018) of the autocorrelation of EEG during movement, Robinson et al. (2017) observed that changes in autocorrelation decay of EEG can also determine the speed of movement.

The ERD has higher sensitivity, and it is likely to give more false positives during the resting state leading to lower overall accuracy (Table 1). The LRTC based classifiers (including LRTC+ERD, ARFIMA and ARFIMA+ERD features) do not have bias toward movement unlike ERD and have similar sensitivity and specificity. Hence, these classifiers are robust and have higher classification accuracy. The movement can be predicted earliest using all the hybrid features containing ARFIMA and ERD up to 1 s before its onset. Thus, using complementary neural correlates of broadband LRTC and narrowband ERD could be beneficial for use in BCI applications.

## 5. Conclusion

We demonstrated broadband LRTCs as novel neural correlates of movement intention in EEG. We propose that the broadband LRTC is the third fundamental neuronal process related to voluntary movement, complementary to the well-known MRCP and narrowband processes such as ERD, providing complementary information about motor command generation.

We identified that the short- and long-range temporal correlations of the broadband EEG estimated from 2 s windows change significantly (*p* < 0.05) during movement intention and execution. Consistently with this, we used ARFIMA(10,*d*,0) to identify ongoing short- and long-range temporal correlations.

Our approach enabled us to predict the movement 1 s before its onset, significantly earlier than the prediction based on ERD. We also obtained significantly higher classification accuracies (*p* < 0.05) than those obtained from ERD. The best classification accuracy was obtained for hybrid ARFIMA (accounting for both short- and long-range temporal correlations) and ERD features (88.3 ± 4.2%). The resulting method offers a robust movement intention detection that might be useful for online BCI.

## Data Availability Statement

The datasets generated for this study can be found in the University of Reading Research Data Archive (http://dx.doi.org/10.17864/1947.117).

## Ethics Statement

The studies involving human participants were reviewed and approved by the ethics committee of the School of Systems Engineering, University of Reading, UK. The patients/participants provided their written informed consent to participate in this study.

## Author Contributions

MW conceptualized the study, designed and conducted the experiments, developed the methodology, analyzed and interpreted the results, created the artwork, and wrote the original manuscript. YH and SN supervised the study, conceptualized the methodology, validated and interpreted the results, and reviewed the manuscript.

### Conflict of Interest

The authors declare that the research was conducted in the absence of any commercial or financial relationships that could be construed as a potential conflict of interest.
