# Supplementary material for: Modeling the Ongoing Dynamics of Short and Long-Range Temporal Correlations in Broadband EEG During Movement
Source: Front Syst Neurosci. 2019 Nov 8;13:66. doi: 10.3389/fnsys.2019.00066 (PMC6856010; doi:10.3389/fnsys.2019.00066)
Supplement: Supplementary file 1 [file Data_Sheet_1.pdf]

frontiers

Supplementary Material

1 Supplementary Table 1

Tables 1A and B show the movement intention classification accuracies, sensitivities and specificities of individual participants for right finger tap and left finger tap using LRTC, ARFIMA, ERD features and their hybrid combinations LRTC and ERD, and ARFIMA and ERD. Classifiers with ARFIMA features gave the highest classification accuracies followed by the classifiers with LRTC features.

Left hand tap															
	LRTC			ARFIMA			ERD			LRTC + ERD			ARFIMA + ERD		
P no.	Acc. (%)	Sen. (%)	Spe. (%)	Acc. (%)	Sen. (%)	Spe. (%)	Acc. (%)	Sen. (%)	Spe. (%)	Acc. (%)	Sen. (%)	Spe. (%)	Acc. (%)	Sen. (%)	Spe. (%)
1	73.00	73.25	72.75	87.25	87.00	87.50	69.13	88.75	49.50*	75.38	76.75	74.00	86.88	86.50	87.25
2	87.55	91.02	84.08	95.31	94.29	96.33	84.29	96.73	71.84	89.59	93.88	85.31	93.88	95.10	92.65
3	73.75	74.25	73.25	91.50	92.75	90.25	69.13	86.25	52.00*	73.50	71.00	76.00	90.00	88.25	91.75
4	81.88	82.00	81.75	87.63	86.50	88.75	76.25	82.50	70.00	79.38	78.00	80.75	90.75	91.50	90.00
5	74.88	83.00	66.75	79.50	77.00	82.00	74.13	95.5	52.75*	78.63	90.75	66.50	87.13	89.25	85.00
6	71.63	77.14	66.12	84.69	80.41	88.98	72.86	76.73	68.98	77.55	80.82	74.29	86.94	88.16	85.71
7	65.00	61.75	68.25	84.63	82.00	87.25	72.75	65.25	80.25	70.63	62.00*	79.25	84.00	86.50	81.50
8	80.38	81.00	79.75	91.00	92.25	89.75	70.00	86.00	54.00*	80.63	79.25	82.00	89.00	89.00	89.00
9	73.25	68.50	78.00	81.38	81.75	81.00	69.88	74.00	65.75	67.25	66.25	68.25	82.50	81.25	83.75
10	66.63	60.50	72.75	89.88	88.00	91.75	63.88	71.25	56.50*	69.00	70.50	67.50	92.38	88.50	96.25
11	87.88	90.25	85.50	94.00	93.75	94.25	81.00	97.75	64.25	88.00	89.00	87.00	94.63	95.00	94.25
12	74.00	72.75	75.25	86.50	86.50	86.50	75.50	94.75	56.25*	77.00	82.50	71.50	88.75	93.25	84.25
13	72.50	73.00	72.00	85.75	87.50	84.00	69.25	59.25	79.25	74.88	70.50	79.25	86.00	81.75	90.25
14	77.38	73.5	81.25	93.75	93.25	94.25	73.63	87.25	60.00*	88.00	87.75	88.25	93.75	96.00	91.50
Mean	**75.69**	**75.85**	**75.53**	**88.05**	**87.35**	**88.75**	**72.98**	**83.00**	**62.95***	**77.82**	**78.50**	**77.13**	**89.04**	**89.29**	**88.79**
SD	6.77	9.10	6.32	4.75	5.51	4.53	5.23	12.12	10.08	6.97	9.66	7.17	3.74	4.57	4.35

Supplementary Table 1A. The peak LDA classification accuracies for the left finger movement vs resting state for all the participants using LRTC, ARFIMA, ERD, LRTC and ERD, and ARFIMA and ERD features. All values except the ones marked by * are significantly above chance level ($p<0.05$).

Right hand tap															
	**LRTC**			**ARFIMA**			**ERD**			**LRTC + ERD**			**ARFIMA + ERD**		
**P no.**	**Acc. (%)**	**Sen. (%)**	**Spe. (%)**	**Acc. (%)**	**Sen. (%)**	**Spe. (%)**	**Acc. (%)**	**Sen. (%)**	**Spe. (%)**	**Acc. (%)**	**Sen. (%)**	**Spe. (%)**	**Acc. (%)**	**Sen. (%)**	**Spe. (%)**
1	75.88	72.00	79.75	90.63	88.75	92.50	67.25	81.50	53.00*	78.75	74.75	82.75	89.88	90.50	89.25
2	84.49	81.63	87.35	91.84	95.51	88.16	84.69	97.14	72.24	92.65	88.98	96.33	95.71	97.96	93.47
3	71.25	76.25	66.25	84.38	87.00	81.75	65.50	72.50	58.50*	71.25	72.00	70.50	81.25	78.00	84.50
4	77.13	76.25	78.00	87.00	84.75	89.25	72.75	81.25	64.25	82.63	84.00	81.25	88.25	88.00	88.50
5	77.50	78.50	76.50	82.13	79.25	85.00	75.00	95.25	54.75	79.00	84.25	73.75	87.50	91.50	83.50
6	77.96	79.59	76.33	92.45	91.43	93.47	71.22	77.96	64.49	86.33	85.71	86.94	93.06	93.88	92.24
7	65.00	70.50	59.50*	77.25	80.50	74.00	69.50	61.25*	77.75	68.38	59.25*	77.50	83.25	82.25	84.25
8	79.00	77.50	80.50	90.38	89.50	91.25	67.25	92.75	41.75*	80.50	84.25	76.75	89.25	87.50	91.00
9	78.50	75.25	81.75	78.63	79.25	78.00	74.88	87.25	62.50	71.63	73.50	69.75	80.63	83.50	77.75
10	74.63	74.75	74.50	85.00	83.50	86.50	62.50	55.50*	69.50	74.75	73.50	76.00	85.63	84.75	86.50
11	89.25	92.50	86.00	88.38	92.25	84.50	80.75	97.50	64.00	89.00	87.25	90.75	92.13	90.00	94.25
12	74.63	76.00	73.25	88.38	90.25	86.50	73.50	87.00	60.00*	75.25	78.25	72.25	89.13	87.25	91.00
13	65.38	66.50	64.25	82.88	83.50	82.25	65.75	72.00	59.50*	72.00	72.00	72.00	82.00	80.50	83.50
14	74.38	72.25	76.50	92.25	91.50	93.00	64.00	58.00*	70.00	78.50	79.50	77.50	88.50	90.25	86.75
Mean	**76.07**	**76.39**	**75.75**	**86.54**	**86.92**	**86.15**	**71.04**	**79.78**	**62.30***	**78.62**	**78.37**	**78.86**	**87.58**	**87.56**	**87.60**
SD	6.40	6.05	7.92	4.98	5.20	5.77	6.40	14.30	9.00	7.14	8.11	7.94	4.56	5.40	4.64

Supplementary Table 1B. The peak LDA classification accuracies for the right finger movement vs resting state for all the participants using LRTC, ARFIMA, ERD, LRTC and ERD, and ARFIMA and ERD features. All values except the ones marked by * are significantly above chance level (p<0.05).

2 Supplementary Figure 1

The figure shows the timing of movement intention detection using classifiers based on LRTC, ARFIMA, ERD features and their hybrid combination. The figure shows the time at which the classification accuracies crossed the significance threshold (chance level) which is 95% confidence level for binary classification (movement vs rest) obtained from the binomial distribution in all the five classifiers.

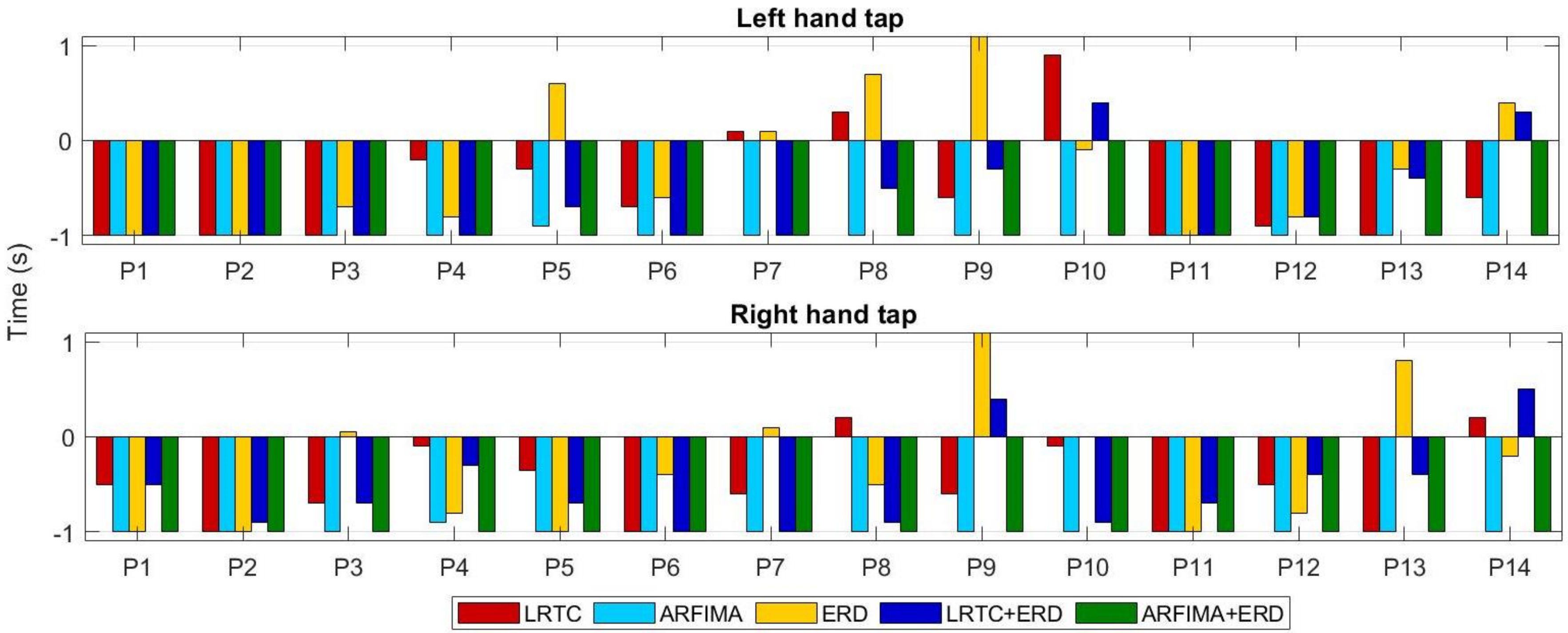

Supplementary Figure 1. The timings of movement intention detection. The timings of movement intention detection when the classification accuracy crossed the significance threshold for right finger tap and left finger tap are shown for all the 14 participants. Timings obtained from classifiers using LRTC, ARFIMA, ERD, LRTC and ERD, and ARFIMA and ERD features are shown.
